# The Vicissitudes of Homophobic Victimization in Adolescence: An Explorative Study

**DOI:** 10.3389/fpsyg.2020.00043

**Published:** 2020-01-22

**Authors:** Ugo Pace, Giulio D’Urso, Lilybeth Fontanesi

**Affiliations:** ^1^Faculty of Human and Social Science, Kore University of Enna, Enna, Italy; ^2^Department of Psychological Sciences, Health and Territory, Università degli Studi “G. d’Annunzio” Chieti – Pescara, Chieti, Italy

**Keywords:** homophobic victimization, adolescents, parental function, mental health, equational structural model

## Abstract

This study investigates the role of parental and peer relationships’ quality on homophobic victimization and possible consequences on mental health during adolescence. Participants were 394 adolescents, (41.6% male and 58.4% female) aged 15–20 years (*M* = 16.55; SD = 0.85), attending the third and fourth classes of public high schools in Italy. Participants completed the Homophobic Bullying Scale to evaluate homophobic victimization toward gays and lesbians or assumed homosexuals, the Symptom Check-list-90 to evaluate mental health, and the Inventory of Parent and Peer Attachment to investigate the quality of peer and parental relationships (in terms of communication, disaffection, and trust). Results show how the quality of peer relationships is not connected with victimization, but the quality of parental relationships is linked with homophobic victimization. Finally, the victimization is connected with anxiety and somatization problems. Theoretical and educational implications were discussed.

## Introduction

Homophobic victimization is a constantly growing phenomenon, especially in scholastic contexts in Western culture (Kosciw, 2004; Poteat and Espelage, 2007; Smith, 2016). These contexts are dominated by a heteronormativity culture (Butler, 2011), which encourages adolescents to victimize those who do not adhere perfectly to the canons of masculinity and femininity imposed by society. Indeed, the victims of homophobic bullying are not only gay and lesbian people but also the alleged ones. The bully using verbal, physical, or cyber violence denigrates and offends, and attempts to annihilate the individuality, the feelings, the attitudes, and the desires of his or her victim (Rivers, 2011; D’Urso et al., 2018). According to Olweus (1991, 1993), an adolescent is victimized when he or she is repeatedly and consistently exposed to negative actions of one or more stronger subjects, thus creating an imbalance of power. Generally, homophobic victimization occurs through the use various form of harm, as offensive epithets (Swearer et al., 2008) or through physical actions (Bontempo and D’Augelli, 2002; Rivers, 2011), as well as through isolation and social exclusion (e.g., Fineran, 2002; Zych et al., 2017; D’Urso and Pace, 2019) and this causes high levels of stress in the victim. In recent years, actions of homophobic bullying have perpetuated through the Internet, resulting in episodes of hate and cyberbullying (e.g., Elipe et al., 2018; Pace et al., 2019a,b).

Homophobic victimization may negatively influence developmental stages and psychosocial well-being (Poteat et al., 2011; Ybarra et al., 2015). The minority stress model (Meyer, 2003) suggests that a victim of homophobic bullying can show negative results related to well-being, specifically implementing states of anxiety and depression related to feelings of inadequacy (Poteat et al., 2011). According to this model, victimization is a form of stress that can affect the adolescent’s adaptive functioning and, therefore, is considered a risk factor related to mental health (D’Augelli, 2002).

Furthermore, the set of homophobic bullying events makes it possible to transform stress and its psychopathological consequences into internalized homophobia (Meyer, 2003). In other words, the adolescent victim of bullying may experience negative feelings that lead him or her to experience feelings of inadequacy about his or her current condition as a gay or lesbian person. According to several studies, victimization is associated with internalization, such as dissatisfaction and the development of symptoms of depression, anxiety, and suicidal ideation (Menesini et al., 2009; Winsper et al., 2012; You and Bellmore, 2012). Furthermore, the literature underlines that even victims of homophobic bullying report levels of depression, anxiety, substance abuse, and suicide attempts (e.g., Baruch-Dominguez et al., 2016; Jones et al., 2018).

The literature also highlights how bullying victims sometimes report poor social skills and coping strategies, which are useful in the development of identity (Waldo et al., 1998), as well as social skills useful for developing sufficiently good social networks, which derive from inadequate parental relations. In line with the attachment theory (Bowlby, 1969; Ainsworth et al., 1978, 2015; Pace et al., 2016), victimization can be a negative outcome resulting from inadequate relational patterns, primarily with parents and subsequently with peers. A “warm social environment” is important in the adolescent socialization process, especially for identity development. Some studies suggest that family relationships and a warm family climate help protect adolescents from the negative outcomes associated with peer victimization (e.g., Bowes et al., 2010). Emotional closeness and time spent with parents diminish during early adolescence (Larson et al., 1996). Although the adolescents struggle to achieve independence and autonomy in adolescence, they always need emotional closeness and “use” their parents to support critical events (e.g., Gutman and Eccles, 2007; Pace et al., 2018).

Consequently, especially for gay and lesbian adolescents, victimization becomes a maladaptive response derived from failed relational patterns. This happens because the gay or lesbian adolescent does not communicate with his or her parents because he or she fears negative reactions, preferring to isolate herself or himself and manage the complexity of a relevant development task, such as coming out or identity development, on his or her own. The literature emphasizes how often victims of bullying are so vulnerable due to the “law of silence,” which strengthens bullies (Elipe et al., 2018). Indeed, especially in homophobic bullying, adolescents report inadequate relational patterns with their parents (Wahl and Metzner, 2012; Labella and Masten, 2018). This happens because often the adolescent does not have the psychological and emotional resources to deal with this problem with his or her parents. Adolescents can, therefore, be dominated by fear and lack of trust because they fear being victimized once again, even by their parents. Furthermore, this event could compromise the disclosure of one’s sexual orientation, and this is not a simple development task.

Therefore, the scarce resources coming from the family context (i.e., poor communication and basic relationships on emotional detachment) can be risk factors connected to homophobic victimization. In line with the attachment theory (Bowlby, 1969; Ainsworth et al., 2015), the adolescent who does not introject internal operational models based on security and affection may not have the resources to face a traumatic moment, such as victimization. The literature, in this sense, suggests how the support and emotional availability of the caregiving context help to prevent traumatic events (Huang et al., 2013).

Furthermore, the literature, in line with the theory of risk factors (Di Blasio, 2005; Passanisi and Pace, 2017), suggests how proximal factors, such as a network of friends, can help the adolescent to resist any stressful or traumatic events and, in general, can help during socialization processes. A class-oriented climate characterized by positivity can reduce episodes of victimization because adolescents are more aware of social rules and respect for each individual and each person (Pace et al., 2019a,b). Therefore, adequate peer relationships can facilitate the reduction of victimization episodes (Hong and Espelage, 2012; Hong and Garbarino, 2012; D’Urso and Pace, 2019). Peer acceptance during adolescence is an important event, influencing a positive development of self and identity: when the experience is negative, as in the case of victimized adolescents, they may not feel supported by their peers because they are not understood and excluded. Therefore, negative episodes of bullying can lead to social isolation, due to the fear of being victimized again. Good relational experiences with peers can lay the foundations for healthy social interactions with adults, but bad ones may be a risk factor for adolescents’ outcomes (Hetherington et al., 1999; Rasalingam et al., 2017).

## The Current Study

Strong models and authors (Bronfenbrenner, 1977; Stern, 1985, 1989) suggest the importance of socio-emotional factors relating to the quality of relationship with parents, as well as the role of peers in the adaptive emotional development of adolescents. These models suggest how emotional availability derived from social relationships can be a protective factor for stressful events that adolescents encounter during their life span. Parents and peers can, therefore, support the adolescent, helping him or her develop those internal emotional resources to deal with development tasks, even the most difficult ones, such as facing or overcoming victimization. With the attempt to broaden the psychological literature, integrating more authoritative models of development, the present study intended to propose a model aimed at explaining homophobic victimization, starting from the parents’ function, and considering the consequences of victimization. As a unique approach in this research, we wanted to analyze not only the aspects related to homophobic victimization in those adolescents who are aware of their homosexual orientation but also the experiences of those who are experimenting, have not yet come out, are unsure or unsecure about their sexuality, or are, because of their behaviors and attitudes, perceived by others as too “feminine” or too “masculine” and teased by peers.

**Figure 1:**
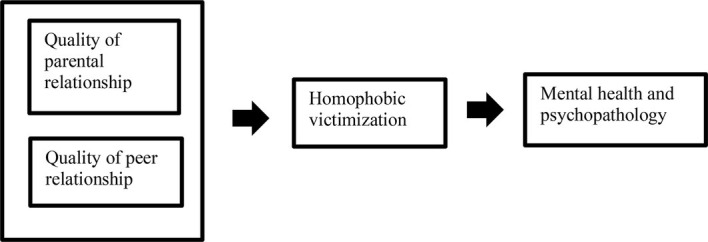
Hypothesized model.

In line with the theory of triadic reciprocal determinism (Bandura, 1978; Swearer et al., 2014), we assume that factors relating to parental bonding and peer relationships can be risk factors related to homophobic victimization. However, in line with the theory of minority stress (Meyer, 2003), we assume homophobic victimization is connected with negative consequences in terms of mental health and psychopathology. Figure 1 summarizes our hypothesized model.

### Method

#### Participants and Procedure

Participants in this study were 394 adolescents, (164 boys–41.6% - and 230 girls–58.4%) aged from 15 to 20 years (*M* = 16.55; SD = 0.85), attending the third and fourth classes of public high schools in Italian cities. About 123 participants (31.2%) attended vocation schools, and 271 participants (68.8%) attended academic schools. We have not investigated sexual orientation because we analyzed episodes of homophobic bullying toward gay and lesbian adolescents and toward presumed homosexuals (i.e., to adolescents who have not yet declared themselves as gay or lesbian). First of all, we contacted the principals of six higher education institutions. They viewed the questionnaires and showed a positive opinion. Then, students’ parents were asked to sign a consent form describing study information and participants’ rights. All parents showed a positive opinion. Data were collected between 2017 and 2018. All questionnaires were administered during lessons, with prior consent. We selected third and fourth classes because we were interested in investigating this phenomenon in adolescence and in classes where peer relationships are already established. Moreover, the literature (e.g., Swearer et al., 2010; Rivers, 2011) underlined more episodes of homophobic bullying in that developmental stage, not only to those who are gay and lesbian but also to those who do not adhere to gender roles. The research was approved by the ethics committee of “Kore” University of Enna. Therefore, all procedures which involved human participants were performed following the ethical standards of the institutional and/or national research committee and with the 1964 Helsinki declaration and its later amendments or comparable ethical standards.

### Measures

#### Demographics

Information used in the current study was gathered on gender, age, and type of school.

#### Victimization

Homophobic Bullying Scale (Prati, 2012). It is a self-report questionnaire designed at measuring homophobic bullying behaviors by pupils, through three perspectives: witness, bully, and victim. In the present study, we considered the victim perspective. The items of this scale concern the victimization actions suffered by participants. The eight items requested adolescents to consider a series of events, such as being marginalized, verbal insulting, or teased in their school contexts because considered gay or lesbian. Below is an example of an item: “during the past 30 days, how often did someone write insulting remarks because you are perceived to be gay or lesbian? Response choices were on a four-point Likert scale ranging from 0 ‘never”’ to 3 ‘more than once a week’.” For the analysis, we used the mean value of the victim perspective scale (*α* = 0.78), related to homophobic victimization through verbal forms and attitudes.

#### Parental and Peer Relationships

The inventory of parent and peer attachment (IPPA; Armsden and Greenberg, 1987). This scale contains a three-part self-report questionnaire that assesses adolescent attachment to mother, father, and peers. It is composed of 25 items for each significant figure. Participants must reply to the questionnaire through a five-point Likert scale (range 1–5), which ranges from 1 “never true” to 5 “always true.” Each individual’s attachment to a specific person (e.g., parents and peers) is assessed *via* three principal subscales (trust, communication, and disaffection). For example, the scale of trust measures the agreement of mutual understanding and respect to significant figure (e.g., peers and parents) and relationship with them (e.g., I trust my parents, My parents understand me), the scale of communication investigates the quality of communication (e.g., I talk to my parents about my concerns; when we discuss things, my parents/peers care about my point of view); the scale of disaffection investigates the emotional detachment felt toward peers and parents (e.g., My parents do not understand what I am going through at this time, I feel angry with my parents/peers). All scales show high-reliability rates (*α*) ranging from 0.80 to 0.90.

#### Internalizing Problems

The Symptom Check-list-90-R (SCL-90-R; Derogatis, 1977) is a 90-item self-report symptom inventory designed to screen for a broad range of psychological problems. Each of the 90 items is rated on a five-point Likert scale of distress, ranging from “`not at all” (0) to “extremely” (4). Subsequently, the answers are combined in nine primary symptom dimensions/scales: Somatization, Obsessive-Compulsive, Interpersonal Sensitivity, Hostility, Depression, Anxiety, Paranoid Ideation, Phobic Anxiety, Psychoticism, and Sleeping Problems. For the present study, we used four of the nine dimensions because they were the ones that, according to the literature, were more connected to the consequences of victimization: the depression scale, composed by 13 items (e.g., Feeling weak or weak), the anxiety scale, composed by 10 items (e.g., Nervousness or internal agitation), the somatization scale, composed by 12 items (e.g., Headaches, Feelings of dizziness, and fainting), and the sleeping problems scale (e.g., Difficulty getting to sleep), composed by three items. The depression scale evaluates feelings of despair, suicidal thoughts, and other cognitive correlates, as well as somatic symptoms related to depressive states (e.g., Feeling everything is an effort). The anxiety scale evaluates the general signs of anxiety such as nervousness, tension, tremors as well as panic attacks and feelings of terror. The scale that concerns somatization problems is reflected disorders that arise from the perception of bodily dysfunctions. While the scale of sleep problems concerns insomnia, disturbed sleep, and early awakening. All four scales show high-reliability rates (*α*) ranging from 0.90 to 0.95. Descriptive statistics about the variables are shown in Table 1.

**Table 1:** Descriptive statistics.

Variables	Mean	SD
Homophobic victimization	1.47	0.15
Parental communication	3.64	0.85
Parental disaffection	2.15	0.81
Parental trust	3.88	0.75
Communication among peers	3.86	0.81
Disaffection among peers	2.21	0.63
Trust among peers	3.11	0.61
Somatization problems	1.01	0.78
Depression	1.10	0.83
Anxiety	1.20	0.86
Sleeping problems	1.00	0.89

### Analysis Plan

At first, the variance analysis was selected to explore gender and school differences concerning homophobic victimization. We have added a model of structural equation (ESM; Hopwood, 2007) in Mplus version 8. Specifically, in this model, we have verified whether communication, trust, and parental and peer disaffection are connected to the victimization suffered. Furthermore, we found whether the victimization suffered could be linked to psychopathological outcomes of anxiety, depression, somatization, and sleep problems. Preliminarily, we had tested this model for males and females, but the effects were almost similar. Therefore, we considered the model for males and females, inserting age, gender, and type of school as control variables in the model.

## Results

**Figure 2:**
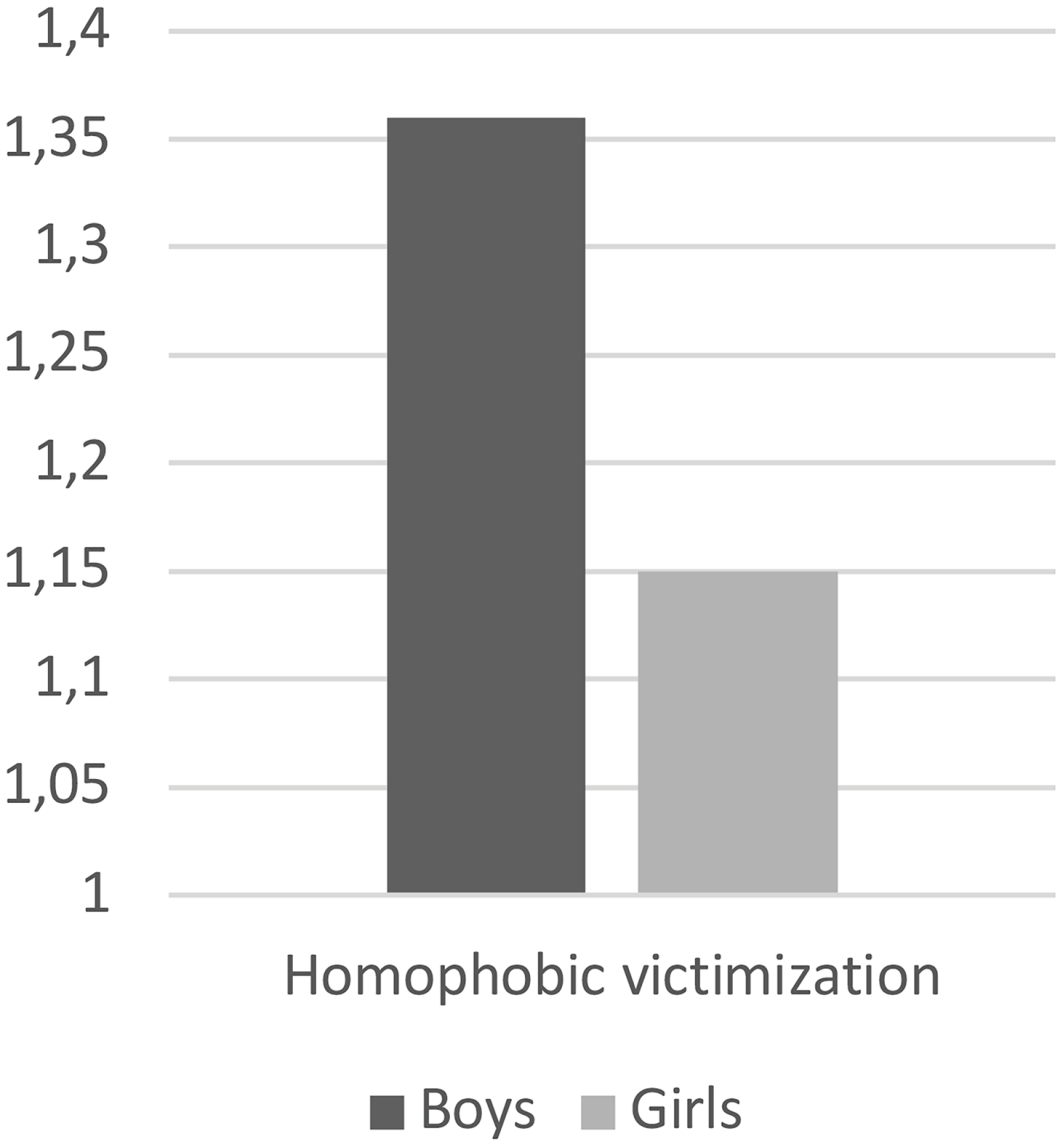
Gender differences on homophobic victimization.

The analysis of variance produces statistically significant effects of gender on homophobic victimization *F*_(1,391)_ = 3.97 with *p* < 0.05. In particular, this result suggests that males (*M* = 1.36; SD = 0.37) report higher levels of victimization than females (*M* = 1.15; SD = 0.33) (Figure 2). No differences emerge regard the type of school.

**Figure 3:**
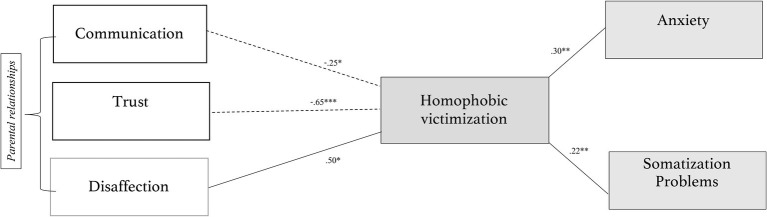
Summary model with significant relationships.

The ESM fits with the data (CFI = 1.00, RMSEA = 0, *χ*^2^(47) = 327.16; *p* < 0.001). The whole model and the explained variation of the model are shown in Figure 3.

In particular, the model suggests that parental disaffection (*β* = 0.50, *t* = 2.22, *p* < 0.05), parental communication (*β* = −0.25, *t* = −1.85, *p* < 0.05), and parental trust (*β* = −0.65, *t* = −3.75, *p* < 0.001) are connected to homophobic victimization. The socio-relational aspect regarding peers [trust (*β* = 0.06, *t* = 0.76), communication (*β* =0.05, *t* = 0.57) and disaffection (*β* = 0.05, *t* = 0.72)] does not produce significant effects.

Furthermore, victimization is related to anxiety (*β* = 0.30, *t* = 2.90, *p* < 0.01) and to somatization problems (*β* = 0.22, *t* = 2.60, *p* < 0.01). Victimization was not significantly related to depression (*β* = 0.10, *t* = 1.01) and sleep disorders (*β* = 0.01, *t* = 0.15). Age, gender, and the types of school do not have any effects on victimization.

## Discussion and Conclusion

The present study aims to investigate how the quality of parental and peer relationships can implement the condition of homophobic victimization, as well as verify the consequences of victimization on the psychological well-being of a group of adolescents in the Italian context. When we talk about victimization, we refer not only to gay and lesbian people but also to the alleged ones. Especially in adolescence in Western culture, victimization is aimed at those who do not adhere perfectly to the stereotypes of masculine and femininity. Indeed, the concept of victimization is broader and not perfectly directed toward those who are positively homosexual.

First of all, our preliminary results suggest how homophobic victimization concerns mainly male adolescents. These data, in line with the literature (Rivers, 2011), may suggest that privileged victims are males, probably because the heterosexist culture and gender stereotypes are things that probably concern more the male gender. Victimizing a man, mostly by diminishing his masculinity, is more common in a heterosexist culture stuck with gender roles (Brown, 2011; Petruccelli et al., 2015). No differences emerged on school types. These data suggest how bullying is a homogeneous phenomenon and may occur in any type of educational institution.

Moreover, the model underlined how parental communication and parental trust are negatively associated with homophobic victimization; in addition, parental disaffection is positively associated with victimization. These data suggest, in line with our theoretical framework (Stern, 1989; Swearer et al., 2014) and with the literature (Bowes et al., 2010), how appropriate relationships with parents built on dialogue on important issues and studded with trust and support can counter homophobic victimization and can operate as protective factors. In particular, for gay and lesbian adolescents, the family may become fundamental because it is the center of the developmental process for identity and may lead to the possible unveiling of a non-heterosexual sexual orientation. In other words, the family can represent the secure basis for facing important development tasks and may act as an emotional container to adequately support the adolescent (Ainsworth et al., 2015), providing him or her with the appropriate resources to relate to peers in contexts dominated by gender biases and stereotypes connected to sexual orientation. The family, in this sense, can be the key factor that breaks the law of silence and helps the adolescent facing any negative event related to sexual orientation. On the contrary, the model suggests how parental disaffection is connected to homophobic victimization. Having feelings of anger and a lack of sufficiently good ties with parental figures, such as a lack of knowledge of the events concerning the adolescent, can make the subject debased and devoid of emotional structures to face homophobic victimization.

Moreover, in this sense, the lack of parental support can make the adolescent feel emotionally poor and unable to find the strength to rebel against homophobic victimization. In addition, the adolescent may be incapable to ask for help from parents. The gay and lesbian adolescent may, for fear of not feeling understood by his or her parents, refuse to “exploit” the parental network, not knowing that in doing so he or she will not be able to face victimization by peers (D’Urso et al., 2017).

Not by chance, indeed, the model suggests how the quality of relations with peers does not work as a risk factor or as a protection factor for homophobic victimization. This can happen because homophobic victimization invalidates the quality of relationships with peers who often see the victim without being emotionally available. Furthermore, adolescents might not be adequately informed about the life stories of the homophobic bullying victim because they do not consider the victims’ experiences and, therefore, do not have the resources to help a peer cope with this event. In this sense, the culture of silence among peers may be so strong to create an obstacle to development. Therefore, the victim of homophobic bullying cannot consider peers as a socio-emotional resource to face critical issues during development (D’Urso et al., 2017).

Finally, in line with Meyer’s (2003) theory, the model underlines how homophobic victimization is connected to negative outcomes on mental health. Indeed, the homophobic victimization is connected to anxiety and somatization problems. Homophobic victimization may be exhibited in adolescence as a state of anxiety characterized by agitation and internal nervousness. Furthermore, the adolescent victim of homophobic bullying can develop psychosomatic problems as tremors, headaches, and nausea. The difficulty of elaborating on a traumatic event, such as homophobic victimization, may be transformed into psychopathological traits that concern more internal psychosomatic problems. We did not find any significant associations between victimization, depression, and sleep disorders, probably because the adolescent manages to disguise the internal emotional sources of his or her depressive states, in addition to not reporting problems related to sleep.

Following the pragmatics of human communication (Watzlawick et al., 2011), functional socio-communicative relationships can make the adolescent stronger when dealing with the vicissitudes related to his or her sexual orientation, and stronger when coping with the psychopathological outcomes of a complex process, such as homophobic victimization. Parent-child and adolescent communication based on sharing and affection turn into the message “your problem exists” and, consequently, “you exist”. Awareness of others and their socio-affective states is the key to coping with homophobic bullying in all its forms. The present study, despite applying the literature, must be considered in light of the limitations present. First of all, the use of self-report questionnaires can surely invalidate social desirability, underestimate one’s symptoms, and evaluate one’s own experiences. Moreover, even the very nature of this cross-sectional study is a limitation. Indeed, emphasizing the cross-sectional nature of the study, it is not possible to create certain links between the variables, but we hypothesize a development model based on the adolescents surveyed in the “here and now” questionnaires. In particular, the relational section is evaluated in relation to past and current schemes; the evaluation of victimization is relative to the last 30 days, and the psychopathological conditions concern the last 7 days. This is why the model is read on the basis of a model A on B and B on C, considering the non-certain causality, but the possibility of a relationship between the variables. Furthermore, even the “does not concern sexual orientation” was considered a limitation of the study. Future studies could include and consider this in the model. However, the study focuses on acts of bullying toward not only gay and lesbian adolescents but also those adolescents alleged to be gay or lesbian and even to people who do not adhere to the canons of masculinity and femininity. The convenience sample also does not allow the results to be generalized to the entire adolescent population. Future studies could, in a longitudinal perspective, analyze homophobic victimization, risk, protective factors, and psychopathological consequences, even in more socio-cultural contexts, thereby, also considering the role of teachers.

In conclusion, the present study suggests significant implications on several fronts. On the psychoeducational level, it is important to implement intervention strategies that promote socio-affective communication not only between parents and children but also among peers. The study highlighted how the school has a key role in order to educate parents and adolescents in the normalization of homosexuality. However, in school contexts, prejudices often survive which can indirectly confirm the bully’s attitudes (e.g., Petruccelli et al., 2015). To prevent victimization would be advisable to start with teachers who then convey educated messages for adolescents and their parents. Moreover, this study emphasizes how parents should need to be trained to recognize internalizing symptoms can be signs of internal distress and consequence of experienced negative episodes, often overlooked. Parents sometimes do not understand deeply that victimization in adolescence may affect the internal emotional states of their daughters and sons. Furthermore, even before this, parents should be educated that various forms of sexual orientation exist and none of them is the result of atypical development.

Prevention interventions aimed at the knowledge of issues concerning sexual minorities and their experiences, as well as awareness-raising, can reduce victimization episodes. Working on prejudices related to gender and sexual orientation is important for creating a more positive social fabric. Prevention of the destruction of prejudice and the education of adolescents should be a central part of a school’s work to affront homophobic bullying since taking steps to prevent bullying makes it easier to avoid every negative episode. It also enables a school to create a rule in which adolescents are clear that bullying acts are unacceptable and will not be tolerated. In this way, the victim can feel recognized, and the bully or bullies can understand the risks he may also face.

In conclusion, this study suggests it is important to monitor the mental health of adolescents because homophobic victimization is not a phenomenon to be overlooked. Bullying actions toward such a profound part of identity and self may lead the adolescent to dysfunctional internalizing emotional states, which during the life span can evolve and result in even more serious psychopathological structures, as these affect the processes of socialization.

## Data Availability Statement

The datasets analyzed in this manuscript are not publicly available. Requests to access the datasets should be directed to ugopax@gmail.com.

## Ethics Statement

The studies involving human participants were reviewed and approved by the Kore University of Enna. Written informed consent to participate in this study was provided by the participants’ legal guardian/next of kin.

## Author Contributions

All authors listed have made a substantial, direct and intellectual contribution to the work, and approved it for publication.

### Conflict of Interest

The authors declare that the research was conducted in the absence of any commercial or financial relationships that could be construed as a potential conflict of interest.
